# Telomerase antagonist imetelstat increases radiation sensitivity in esophageal squamous cell carcinoma

**DOI:** 10.18632/oncotarget.14618

**Published:** 2017-01-13

**Authors:** Xuping Wu, Jing Zhang, Sijun Yang, Zhihui Kuang, Guolei Tan, Gang Yang, Qichun Wei, Zhigang Guo

**Affiliations:** ^1^ The Second Hospital of Nanjing Affiliated to Medical School of Southeast University, Nanjing 210003, China; ^2^ Jiangsu Key Laboratory for Molecular and Medical Biotechnology, College of Life Sciences, Nanjing Normal University, Nanjing 210023, China; ^3^ ABSL-3 Laboratory at The Center for Animal Experiment and State Key Lab of Virology, Wuhan University, Wuhan 430071, China; ^4^ Department of Radiation Oncology, Second Affiliated Hospital, Zhejiang University School of Medicine, Hangzhou 31009, China

**Keywords:** esophageal squamous cell carcinoma, telomerase, radiosensitization, apoptosis

## Abstract

The morbidity and mortality of esophageal cancer is one of the highest around the world and the principal therapeutic method is radiation. Thus, searching for sensitizers with lower toxicity and higher efficiency to improve the efficacy of radiation therapy is critical essential. Our research group has previously reported that imetelstat, the thio-phosphoramidate oligonucleotide inhibitor of telomerase, can decrease cell proliferation and colony formation ability as well as increase DNA breaks induced by radiation in esophageal cancer cells. Further study in this project showed that imetelstat significantly sensitized esophageal cancer cells to radiation *in vitro*. Later study showed that imetelstat leads to increased cell apoptosis. We also measured the expression level of several DNA repair and apoptosis signaling proteins. pS345 CHK1, γ-H2AX, p53 and caspase3 expression were up-regulated in imetelstat treated cells, identifying these factors as molecular markers. Mouse *in vivo* model using imetelstat at clinically achievable concentrations and fractionated irradiation scheme yielded results demonstrating radiosensitization effect. Finally, TUNEL assay, caspase 3 and Ki67 staining in tumor tissue proved that imetelstat sensitized esophageal cancer to radiation *in vivo* through promoting cell apoptosis and inhibiting cell proliferation. Our study supported imetelstat increase radiation sensitivity of esophageal squamous cell carcinoma through inducing cell apoptosis and the specific inhibitor of telomerase might serve as a potential novel therapeutic tool for esophageal squamous cell carcinoma therapy.

## INTRODUCTION

Esophageal carcinoma is a common malignant tumor of digestive system with the mortality rate ranking the sixth in the world [1]. Surgical resection is preferred for patients with early esophageal cancer. However, patients in clinical mainly suffer from advanced esophageal cancer [2–3] which is often accompanied by lymph node metastasis or other complications. Radiation is one of most important and effective therapeutic methods for esophageal cancer. However, esophageal cancer belongs to the moderately sensitive tumors to radiotherapy. The 3-year overall survival rate has been reported to be only about 20% after radiotherapy alone while the recurrence rate is as high as 60%–80% [4]. Although increasing the irradiation intensity could improve the therapeutic effect, the normal tissues might be also damaged and radioresistance might be another major challenge resulting in locoregional recurrence and metastasis [5]. Thus, it is essential to develop radiation sensitizer with high efficiency and low toxicity for the treatment of esophageal cancer [6].

It has been extensively reported that targeting telomerase is effective for cancer treatment [7] and the most direct way is to inhibit the activity of telomerase is targeting telomerase RNA (hTR) or reverse transcriptase protein subunit (hTERT). Imetelstat, a 13-mer oligonucleotide which is covalently modified with lipid extensions, competitively suppresses enzymatic activity of telomerase [8]. It has been found to target the template region of hTR and serve as an oligonucletide template antagonist [9]. Covalently bonded liposomes are prone to be uptaken by cell, which ensures a better and reliable bioavailability. Meanwhile, thio-phosphoramidate internucleoside linkages in imetelstat provide a longer half-life for the inhibitor *in vivo* [10–11]. Furthermore, it has been reported that telomerase activity was remarkably elevated and inhibition of telomerase blocks proliferation of esophageal adenocarcinoma cells both *in vitro* and *in vivo* [12], while there was no report focusing on esophageal squamous cell carcinoma.

Radiation produce large amounts of free radicals which further leads to DNA breakage. DNA double strand breaks (DSBs) is not only the most serious injury caused by radiation, but also the basis of radiation to kill tumor cells [13]. Once DSBs is induced, cells themselves react quickly and activate DNA damage responses which recruit large amounts of protein such as ATM, γ-H2AX, p53 to sense, amplify and transduce DNA damage signal rapidly [14]. Eventually, cells respond to these signals to protect themselves, including cell cycle checkpoints, regulation of gene expression and cell apoptosis.

In the present study, we investigated the increased radiosensitization effect of imetelstat on esophageal squamous cell carcinoma *in vitro* and *in vivo*. The effect of imetelstat on proliferation and radiosensitization was correlated with its ability to increase cell apoptosis.

## RESULTS

### Imetelstat enhanced Kyse410 and Kyse520 sensitivity to radiotherapy

**Figure 1:**
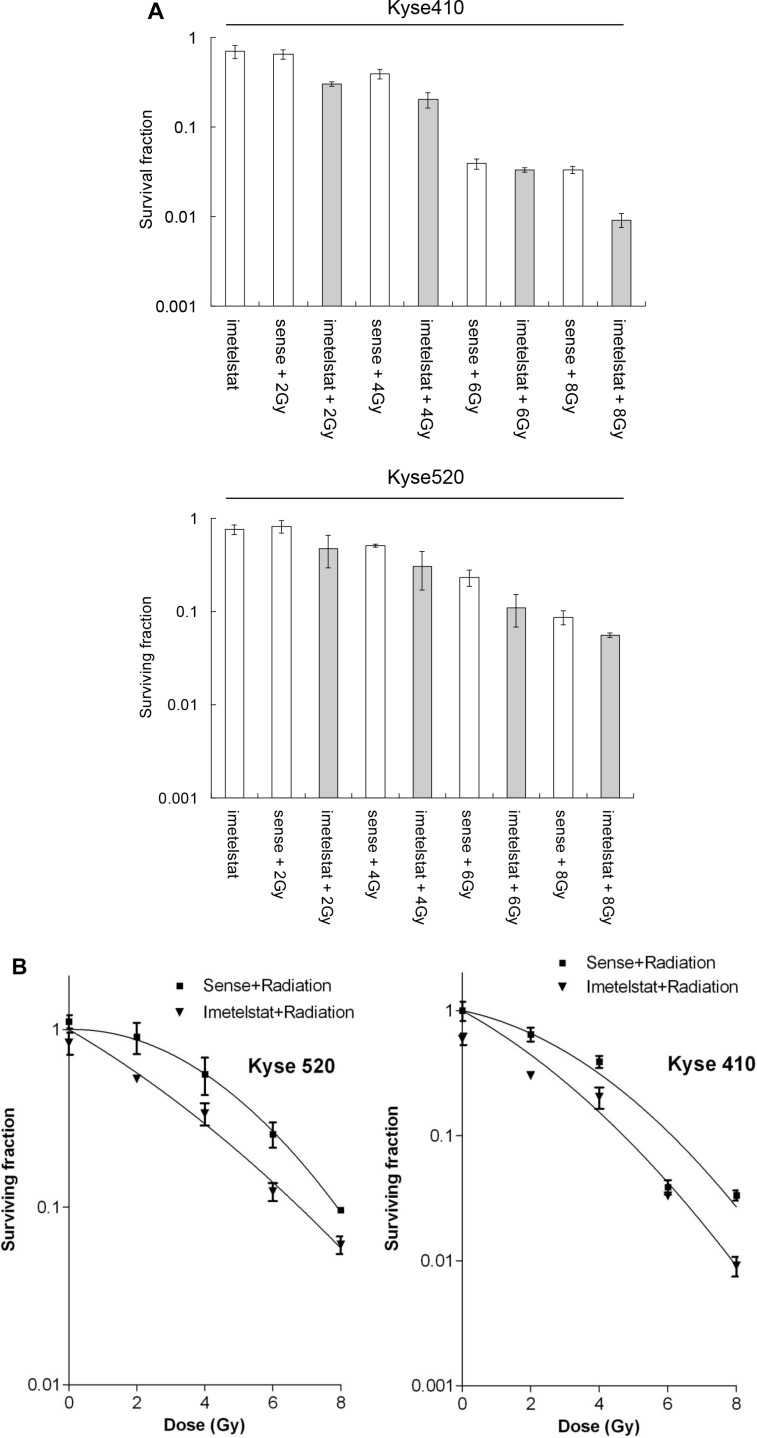
Imetelstat radiosensitizes Kyse410 and Kyse520 cells by using clonogenic cell survival following imetelstat and/or X-ray irradiation (2, 4, 6 and 8 Gy) **(A)** shows survival fraction as a function of imetelstat, radiation dose and their combination. **(B)** shows survival plots as a function of radiation dose with or without imetelstat. Survival data were fitted to S = exp (-αD-βD2) where D is the dose in Gy and α and β are fitting parameters, n ≥ 6, Error bars represent standard deviation. All imetelstat treatment had P-values less than 0.05 except imetelstat + 6 Gy treatment for Kyse410.

Prior investigations have revealed that inhibitor imetelstat treated esophageal squamous cells is able to increase the number of double-strand breaks induced by high energy X-Ray [9]. To further study the radiosensitization of imetelstat on esophageal squamous carcinoma cells, the present study was carried out on the basis of previous researches. As shown in Figure 1A, exposure to imetelstat alone resulted in 69.7% cell survival for Kyse410 and 75.9% cell survival for Kyse520 compare to sense treatment. Kyse410 and Kyse520 cells were exposed to radiation of 0, 2, 4, 6 and 8 Gy X-ray in the presence or absence of imetelstat to study cell survival. Cell survival after 2, 4, 6 and 8 Gy irradiation of Kyse410 was 64.7%, 39.1%, 3.9% and 3.3%, whereas the expected additive survival should be 45.0%, 27.2%, 2.7% and 2.3%. However, the achieved cell survival after combination was less, 30.1%, 20.3%, 3.3% and 0.9%. For Kyse520, the expected additive cell survival is approximately 61.4%, 38.3%, 17.4% and 6.5% theoretically. Whereas, the achieved cell survival of Kyse520 exposed to 2, 4, 6 and 8 Gy irradiation and imetelstat combination was 47.6%, 30.2%, 11.0% and 5.5%. Figure 1B showed the survival curves of Kyse410 and Kyse520 after exposure to X-ray combined with the treatment of 5 μM imetelstat, which reflected the radiosensitizing effect. As indicated, imetelstat treated cells were normalized to sense treated controls, the figure only reflected the raidosensitizing effect. We observed that imetelstat could radiosensitize both Kyse410 and Kyse520 cells, the obtained SERs were 1.9 and 1.6, respectively.

### Imetelstat increases cell apoptosis of Kyse410 and Kyse520 cells

**Figure 2:**
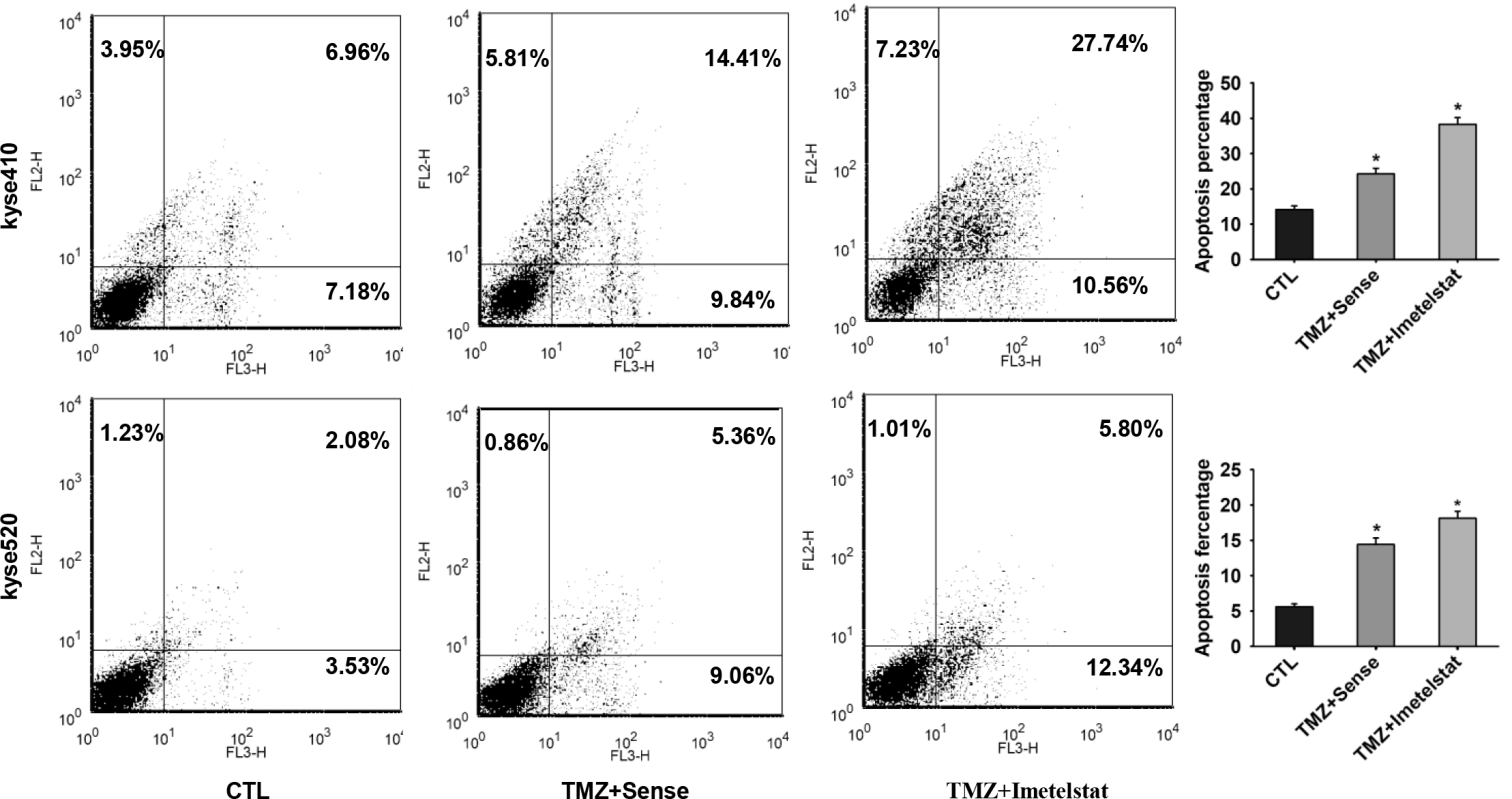
Imetelstat increases cell apoptosis of Kyse410 and Kyse520 cell Kyse410 and Kyse520 cells were incubated and treated with 5 μM sense or imetelstat for 72 h and at the same time exposed to 10 μM TMZ. Results of early and late apoptosis were added together to calculate the total amount of apoptosis. Values represent the mean±SD and are representative of three independent experiments. *p < 0.05, statistical significance was established by t test.

Apoptosis, a mode of cell death in response to radiation, is an important method for cancer therapy. To test whether Kyse410 and Kyse520 cell apoptosis was enhanced by imetelstat, annexin V - FITC staining were applied. Temozolomide (TMZ) can form DNA double strand breaks and induce cell apoptosis. In later studies, TMZ was selected to mimic the function of radiation *in vitro*. As shown in Figure 2, TMZ significantly increased apoptosis percentage for both cell lines compared with the control group (*p <* 0.05). Apoptosis rate for Kyse410 and Kyse520 cells was approximately 24% and 14% with the exposure to TMZ/sense. Imetelstat administration increased the apoptosis percentage to 38% for Kyse410 and 18% for Kyse520, significantly higher than those treated with sense (*p <* 0.05).

**Figure 3:**
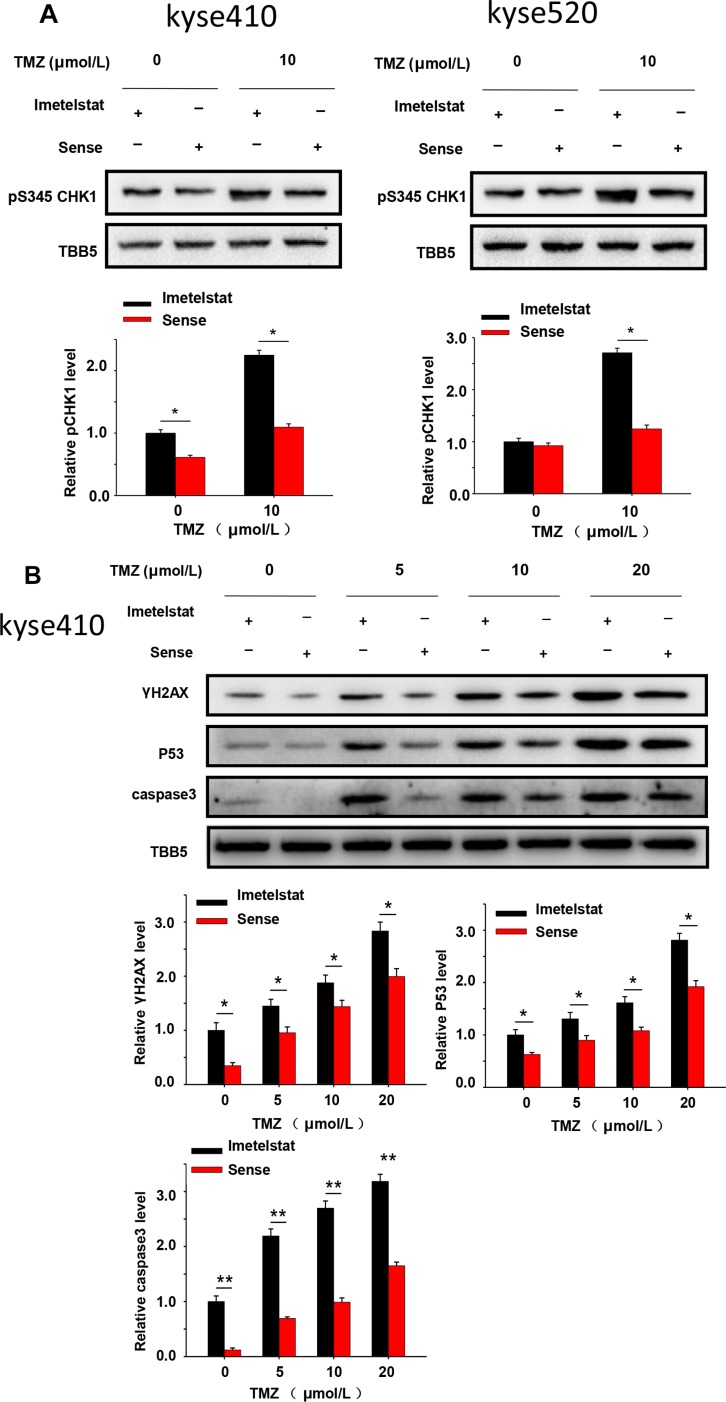

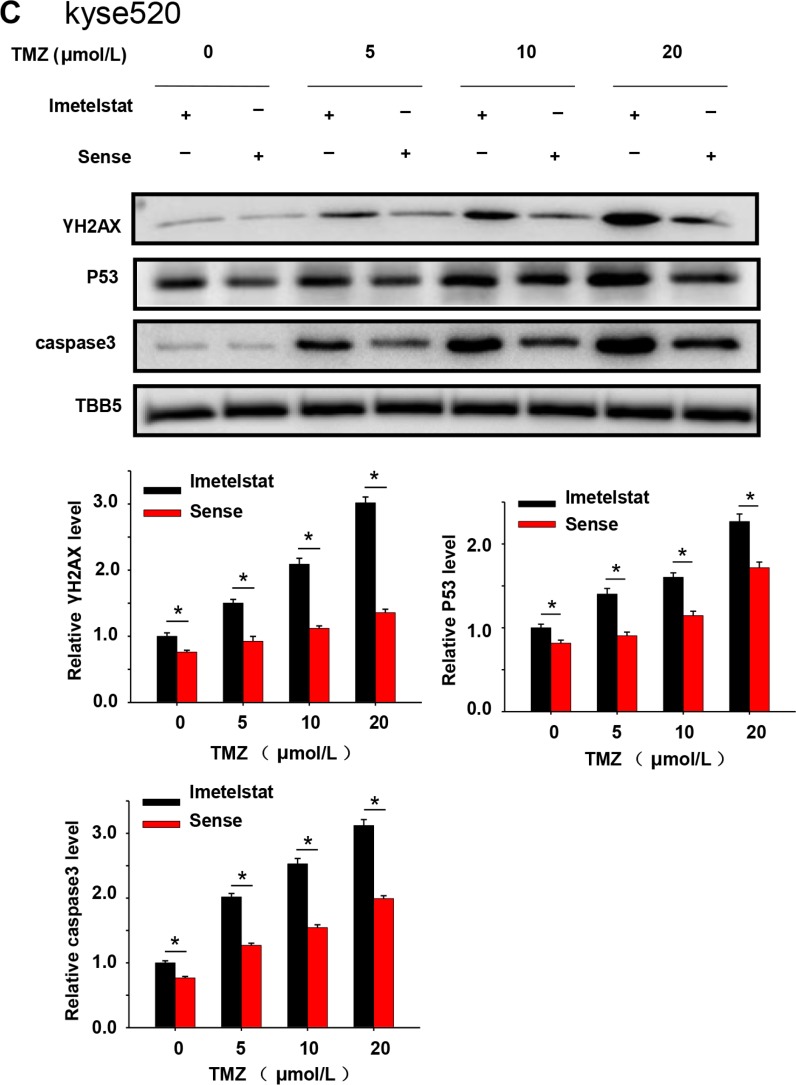
DNA repair and apoptosis signaling protein were upregulated by the treatment of imetelstat in Kyse410 and Kyse520 cells **(A)** Kyse410 and Kyse520 cells were treated with 5 μM imetelstat or 5 μM sense for 72 h and meanwhile exposed to 10 μM TMZ. The expressions of phosphorylation of CHK1 at S345 were detected, taking the expression of TBB5 as inner control. (B and C) Kyse410 and Kyse520 cells were treated with 5 μM imetelstat or 5 μM sense for 72 h together with TMZ (0, 5, 10, 20 μM). Expression levels of γ-H2AX, p53 and caspase 3 were detected by immunoblotting. The intensity of each protein band was quantified using the BandScan software (Glyko) and normalized against tublin. *represents statistical significant (*p* < 0.05).

Cells react to DSBs by triggering the DNA damage checkpoint response, which arrests cell-cycle progression until the DNA damage has been removed. CHK1 is a multifunctional protein kinase that plays essential roles in cell survival and cell cycle checkpoints. As shown in Figure 3A, Kyse cells were treated with 10 μM TMZ and showed increased phosphorylation of CHK1. Furthermore, imetelstat treatment could increase CHK1 phosphorylation compare with sense treatment. The magnitude and rate of DNA double-strand breaks repair were assessed by the analysis of γ-H2AX using western blot. Cell treated with TMZ over a dose range of 5, 10 and 20 μM showed increased γ-H2AX in the imetelstat-treated samples for both Kyse410 and Kyse520 cells (Figure 3B and 3C), while the relative protein levels in Kyse410 cell was higher than that in Kyse520 cells. Human tumor suppressor gene p53 is known to be implicated in DNA repair and induce cell apoptosis. As illustrated in Figure 3B, the expression of p53 in Kyse410 cells was up-regulated with the treatment of imetelstat. Besides, caspase family plays a critical important role in mediating the process of apoptosis, wherein caspase3 is key execution molecule. It was also found that the expression of caspase3 in Kyse410 and Kyse520 cells were up-regulated with the treatment of 5 μM imetelstat (Figure 3B and 3C).

### Imetelstat sensitizes esophageal cancer cells to radiation *in vivo*

**Figure 4:**
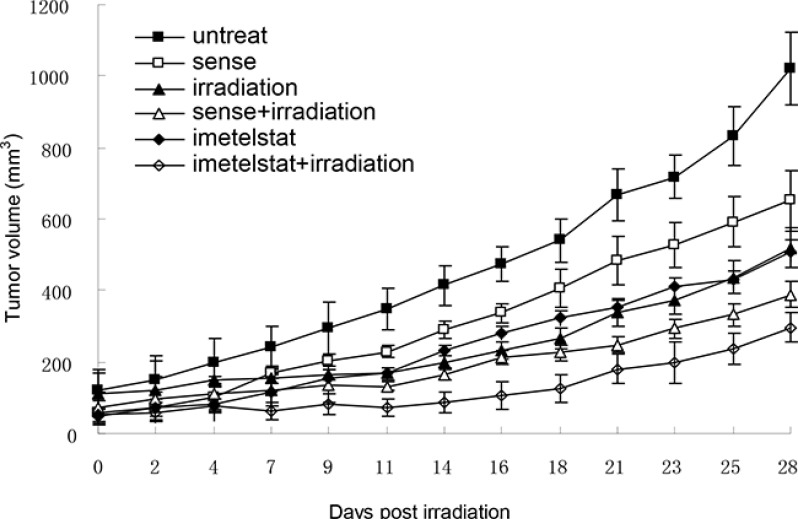
Imetelstat sensitizes esophageal cancer cell Kyse520 to radiation *in vivo* Kyse520 cells were pretreated with imetelstat or sense for 7 weeks before injected into nude mice to form tumors. When the size of tumor reached approximately 50 mm3, mice were randomly divided into 6 different groups, including untreated group, radiation group, sense group, imetelstat group, sense + radiation group and imetelstat + radiation group. Tumors in nude mice were subjected to 2 Gy of irradiation for 5 consecutive days to mimic a fractionated weekly scheme. The sizes of tumor were measured 3 times per week to obtain cell growth curve. At days 13, 31 and 45 postinjection, imetelstat were injected intraperitoneally to ensure inhibition of telomerase activity. Data are presented as mean tumor volume ± SE. Imetelstat with irradiation significantly decreased tumor growth compared with mice receiving irradiation alone or irradiation with sense (*p* < 0.05).

To study whether telomerase inhibition and telomere dysfunction was associated with a progressive impairment in the cell growth *in vivo* as well as *in vitro*, the tumor growth curve was pictured. Kyse cells were pretreated with imetelstat and tumors in nude mice were subjected to 2 Gy of irradiation for 5 consecutive days. As seen in Figure 4, the volume of tumor in nude mice treated with X-ray exhibited a decrease in contrast with the untreated and sense group. As a telomerase inhibitor, imetelstat itself could significantly inhibit the growth of tumor compared with those in the sense group. More importantly, Kyse520 in imetelstat/radiation group showed a lag in tumor growth compared with mice receiving 2 Gy of irradiation alone or irradiation with sense, which confirmed that imetelstat made tumor more sensitive to radiotherapy.

### Imetelstat increases cell apoptosis and decreases cell proliferation *in vivo*

**Figure 5:**
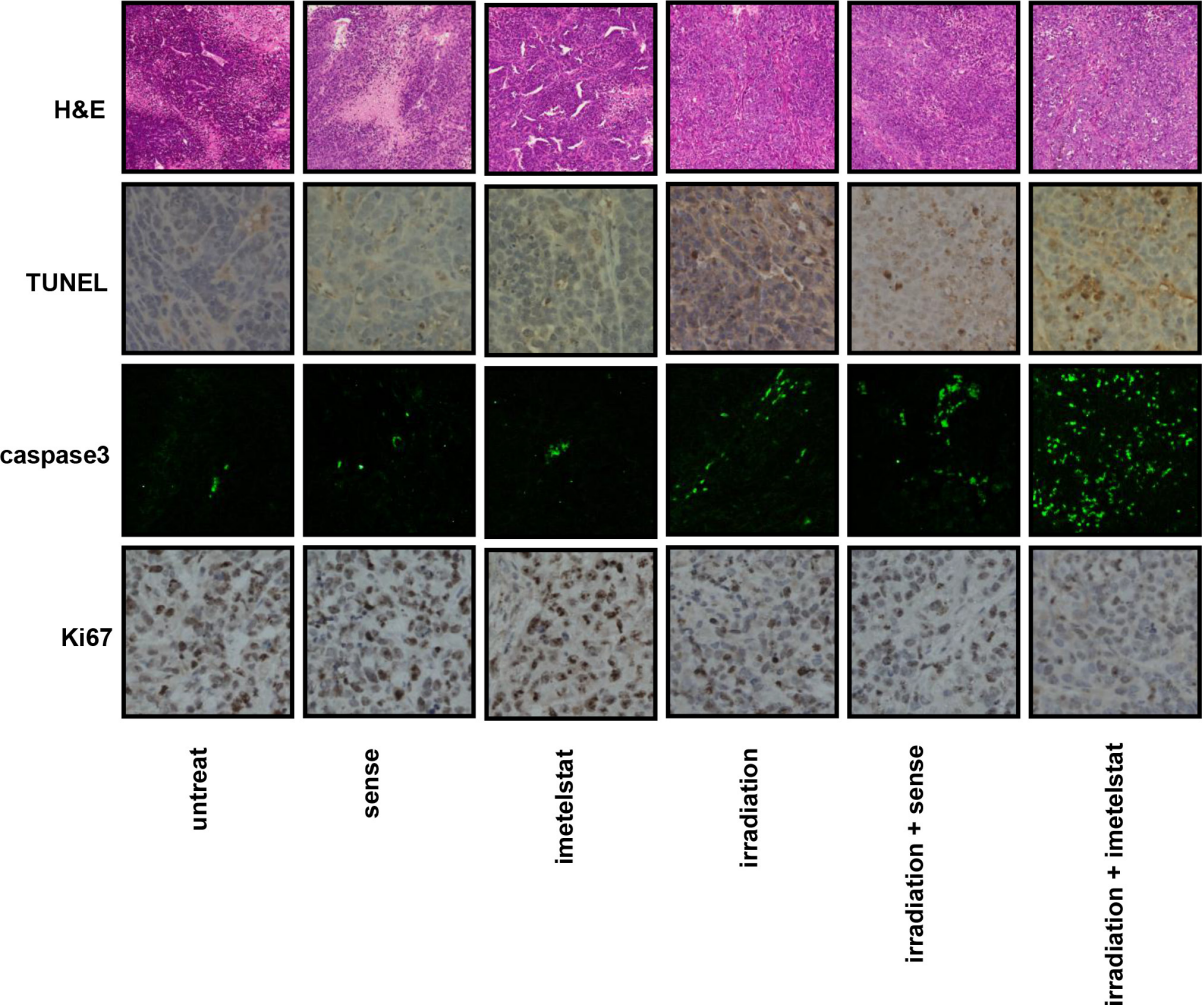
Imetelstat increases cell apoptosis and decreases cell proliferation *in vivo* Pathological changes of esophageal squamous carcinoma were evaluated by H&E staining. Apoptotic cells were labeled by TUNEL assay and further evaluated by immunofluorescence staining of caspase 3. Imetelstat promoted radiation-induced cell apoptosis compared with radiation-treated tumor. The cell proliferation was evaluated by Ki67 staining in tumor tissue. Imetelstat combined with radiation showed less Ki67 expression compared to other treatment groups.

Pathological changes of esophageal squamous carcinoma were evaluated by H&E staining. As seen in Figure 5, tumor samples from the untreated group were tightly aligned with a large blue-hued nucleus. Besides, samples from mice subjected to imetelstat alone displayed more vacuoles in contrast with those in the sense group. Tumor isolated from mice in the imetelstat/radiation group showed condensed cytoplasm and desmosome complexes, fragmented nuclear, separated cells in comparison with those in the sense/radiation group, suggesting the potential sensitizing effect.

Apoptotic cells were labeled by TUNEL assay as shown in Figure 5. Although TUNEL staining for tumor treated with sense or imetelstat showed relatively small number of apoptotic cells, the enhanced staining intensity in imetelstat group was still stronger than that in the sense group. Moreover, tumor exposed to the combination of radiation and sense also showed less apoptosis staining than those subjected to imetelstat/radiation. The apoptosis induced by imetelsat and radiation was further analyzed by using caspase3 immunofluorescence staining. The expression of caspase3 in radiation-treated tumor *in vivo* was up-regulated, while imetelstat promoted radiation-induced cell apoptosis as reflected by the further up-regulated level of caspase3 (Figure 5). More importantly, the expression of caspase3 in imetelstat/radiation group was much higher than that in the sense/radiation group, which implies more apoptosis. Tumor subjected to sense or imetelstat only showed almost no expression of caspase3, which confirmed that imetelstat was not toxic to mice *in vivo* and the principal function of it was enhancing tumor sensitivity to radiation therapy.

The expression of Ki67 is indispensable in cell proliferation. Radiation-treated mice showed the down-regulation of Ki67 and imetelstat enables tumor more sensitive to radiation treatment, which was revealed by weaker expression of Ki67 (Figure 5). As observed in Figure 5, the staining of Ki67 in sense or imetelstat treated group was stronger compared with that exposed to radiation. More importantly, tumor pretreated with sense combined irradiation displayed higher level of Ki67 compared with that in the imetelstat/radiation group, which indicated radiosensitization effect of imetelstat. These findings suggested that imetelstat inhibited tumor growth in mice subjected to radiation was associated with the enhanced apoptosis and suppressed cell proliferation in cancer cells.

## DISCUSSION

Telomerase activation is considered as a key step in cell immortalization and tumorigenesis [15]. It has been reported that positive rate of telomerase activity is 80% to 90% in squamous cell carcinoma, which is much higher than those in the surrounding normal tissues [16]. This discrepancy between cancer and normal cells provides a considerable therapeutic window for telomerase suppression-based therapy.

Our prior work has verified that imetelstat blocks telomerase activity in esophageal cancer cells, decreases cell proliferation and colony formation ability. More importantly, our research group has reported imetelstat increases radiation induced DNA breaks, which provided theoretical foundation for further researches [9]. In this study, we found that long-term imetelstat treatment could radiosensitize Kyse cells in both vitro and *vivo*. This is consistent with one study on breast cancer cells, in which long-term treatment of imetelstat showed radiosensitization activity *in vitro* colony formation assay and *in vivo* tumor growth assay [17]. Further analysis showed that SER was larger for Kyse410 than Kyse520, 1.9 and 1.6 respectively. These results indicated that Kyse520 is more radioresistant than Kyse410 cells, consisting with our previous results that radiation-induced DSB foci in Kyse410 are more in number and larger in size compared with the DSB foci in Kyse520 cells [9].

Multiple DNA damage repair pathways are implicated in maintaining the genetic integrity of a cell after its exposure to radiation. Wong et al. reported that telomerase deficient mouse developed a radio-sensitivity syndrome because of the delayed DNA break repair [18]. Our previous study reported that treatment of imetelstat and radiation showed synergistic increase and prolonged higher expression of DSB foci compared to cells treated with sense/radiation [9]. In this study, western blot results showed that increased expression of phospho-CHK1 and γ-H2AX in imetelstat treatment group which reflect the magnitude and rate of DSB repair. Our results are consistent with one report in glioblastoma cells that increased levels of γ-H2AX after imetelsat treatment [19]. Cells react to DSBs by triggering the DNA damage signaling, which will further activate intracellular pathways including apoptotic signaling [20–22] One our previous research reported that 17-AAG sensitize esophageal squamous cells to radiation by inhibiting cell proliferation and promote cell apoptosis [23]. Ding et al. reported that sunitinib increased radiation induced DNA double-strand breaks and promoted the apoptosis of human esophageal squamous cell carcinoma cells [24]. Our study showed that imetelstat combined TMZ which mimic the function of radiation could significantly increase cell apoptosis compared with sense/TMZ treatment (Figure 2). Western blot demonstrated that imetelstat treatment could increase expressions of caspase3 and p53, which promoted cell apoptosis and reflected increased DNA repair signaling (Figure 3B and 3C). Apoptotic cells labeled by TUNEL assay in tumor tissue were increased in imetelstat treatment group which is further proved by caspase3 immunofluorescence staining (Figure 5).

Although *in vitro* data provide an important preliminary background to advance the potential use of imetelstat, these results must be confirmed and validated *in vivo*. The clinical application of therapeutic irradiation relies on fractionated does of radiation that are given daily for several weeks. In our study, we extended *in vivo* results to a fractionated weekly scheme consistent with clinical treatment protocols. Our experiments demonstrated that imetelstat combine with radiation induce tumor growth delay compared to imetelstat treatment or sense/radiation treatment. The results comfirmed that imetelstat can radiosensitize esophageal cancer *in vivo*. As a marker of cell proliferation, antigen Ki67 was also detected to confirm the tumor growth delay induced by imetelstat treatment.

In conclusion, imetelstat increases radiation sensitivity in esophageal cancer by inducing cell apoptosis and decreasing cell proliferation. *In vitro* and *vivo* results support imetelstat as a promising adjuvant cancer treatment in combination with radiation therapy.

## MATERIALS AND METHODS

### Main reagents and kits

The human ESCC (esophageal squamous cell carcinoma) cell Kyse410 and Kyse520 (Deutsche Sammlung von Mikroorganismenund Zellkulturen GmbH, Braunschweig, Germany) were cultivated in PRMI-1640 medium at 37°C in a humidified 5% CO2 incubator, supplemented with 10% fetal bovine serum, 100 U penicillin and streptomycin. Primary antibodies against γ-H2AX, p53, caspase3, and pS345-CHK1 were purchased from Cell Signaling Technology (Beverly, MA). Anti-Ki-67 and tublin antibodies was purchased from Santa Cruz Biotechnology (Santa Cruz, CA). PRMI-1640 medium was from GIBCO and fetal bovine serum (FBS) was purchased from Hyclone (Logan, UT, USA). Penicillin and streptomycin were purchased from Amresco Inc (Solon, OH).

### Radiosensitization experiment

Kyse410 and Kyse520 were pretreated with imetelstat and sense at a concentration of 1 μM for 40 days. During this time, cells were passaged every three days and fresh antagonist was added. After that, cells were exposed to radiation of 0, 2, 4, 6 and 8 Gy of 6 MeV X-ray at room temperature, respectively. To assay clonogenic cell survival, cells were trypsinized and plated onto 10-cm tissue culture dishes. Cells were incubated for 10–12 days, fixed with ethanol and stained with haematoxylin. Cells able to form a colony of at least 50 cells were considered as clonogenic cells. Survival data were fitted to S = exp (-αD-βD^2^) where D is the dose in Gy and α and β are fitting parameters. Radiosensitivity was quantified as area under curve (AUC) and the effect of imetelstat on radiosensitivity is presented as sensitizer enhancement ratio (SER), defined as AUC _control_/AUC _treated_.

### Apoptosis assay

Kyse410 and Kyse520 cells were incubated and treated with 5 μM imetelstat or 5 μM sense for 72 h and together with 10 μM temozolomide (TMZ). Cells were harvested, washed, resuspended in PBS and then stained with the Annexin V/PI Cell Apoptosis Detection Kit (Franklin Lakes, NJ) according to the manufacturer's instructions. Data acquisition and analysis were conduct with a Becton–Dickinson FACS Calibur flow cytometer using Cell-Quest software (BD Biosciences, Franklin Lakes, NJ).

### Western blot

Kyse410 and Kyse520 cells were incubated and treated with 5 μM imetelstat or 5 μM sense for 72 h and meanwhile exposed to increased concentration of TMZ (0, 5, 10, 20 μM). Subsequently, proteins in Kyse410 and Kyse520 cells were extracted with RIPA lysis buffer containing a protease inhibitor cocktail, PMSF for 30 min on ice respectively and then centrifuged at 12000 rpm for 5 min at 4°C. Total protein concentration in supernatant of cell lysates was determined by Pierce BCA protein assay kit (Thermo, Massachusetts, USA). Then we used γ-H2AX, p53, caspase3 and pS345-CHK1 Abs in the following SDS-PAGE and Western blot detection. Antigens were detected after incubation with an HRP-conjugated secondary antibody followed by visualization using an ECL detection system. The intensity of each protein band was quantified using the BandScan software (Glyko) and normalized against tublin in the same samples blotted by anti-tublin antibody.

### Animals care and experimental protocol

All animal maintenance and experimental procedures were carried out in accordance with the National Institutes of Health Guide for the Care and Use of Laboratory Animals. Male nude mice were purchased from Beijing HFK bioscience Company Limited. Mice were maintained in an air-conditioned pathogen-free room under conditions of controlled lighting of 12 h light/day. Five million Kyse520 cells cultured were cultured for 7 weeks adding 1 μM imetelstat or sense. Esophageal squamous Kyse520 cell suspension was injected subcutaneously into right hind limb groin of nude mice to induce the formation of tumor. When the size of tumor reached approximately 50 mm^3^, mice were randomly divided into 6 different groups, including untreated group, radiation group, sense group, imetelstat group, sense + radiation group and imetelstat + radiation group. After that, tumors in nude mice were subjected to 2 Gy of irradiation for 5 consecutive days, while other parts were protected by lead. The sizes of tumor were measured 3 times per week to obtain cell growth curve. Mice were sacrificed 50 days later and tumors were dissected subsequently for further analysis. At days 13, 31 and 45 postinjection, inhibitor imetelstat (30 mg/kg) and control sense (30 mg/kg) were injected intraperitoneally to ensure inhibition of telomerase activity.

### TUNEL assay

DNA fragments in tissue sections were detected using TUNEL assay kit (BD Pharmingen, CA) according to the manufacturer's instructions. Briefly, the enzyme terminal deoxynucleotidyl transferase (TdT) was used to incorporate digoxigenin-conjugated dUTP at the ends of DNA fragments. The signal of TdT-mediated dUTP nick-end labeling was then detected using an anti-digoxigenin antibody conjugated with peroxidase. TUNEL slides were photographed with a confocal laser scanning microscope (Fluoview FV1000, Olympus, Tokyo, Japan). Positive staining of cells within each slide were measured and expressed as a ratio of apoptotic cells to total number of tumor cells.

### Immunohistochemistry and immunofluorescence assay

Tumor tissues were fixed in 10% (V/V) neutral buffered formalin solution for at least 24 h at room temperature, dehydrated in graded ethanol, cleared in xylene and embedded in paraffin. Perpendicular tumor sections (4 μm thickness) were mounted on glass slides and de-waxed. Then, tissue sections were prepared for Hematoxylin-Eosin (HE) staining. Pathological changes were observed by light microscopy. The expression of caspase3 in tumor was detected by immunofluorescence assay. Tumor tissues was processed and mounted on glass slides. Briefly, sections were pre-incubated with 1% Triton X-100 and 10% bovine serum albumin (BSA) in phosphate buffered saline (PBS) for 1 h at room temperature. After that, samples were incubated with specific antibodies against caspase3 for 2 h at room temperature. Fluorescent secondary antibodies were performed for another 1 h at room temperature according to the manufacturer's instructions. The expression of Ki67, proliferation cell-associated nuclear antigen in tumor tissues, was assessed by immunohistochemistry. The paraffin sections were heat fixed, deparaffinized in xylene, rehydrated by graded ethanol to distilled water, boiled in sodium citrate buffer and incubated in 3% hydrogen peroxide after cooling. Each slide was blocked with 3% BSA at room temperature prior to the incubation with primary antibody overnight at 4°C, secondary antibody for 20 min at 37°C and streptavidin-HRP 20 min at 37°C, respectively. Samples were then stained with DAB and counterstained with hematoxylin. After dehydrating and drying, the sections were mounted with neutral gum.

### Statistical analysis

All data were presented as means ± standards deviation (SDs). One-way analysis of variance (ANOVA) followed by Tukey multiple comparison test was performed to evaluate differences between groups. The values were considered statistically significantly different at *p* < 0.05.
